# Culling Reasons in Industrial Dairy Farms of Western Tehran Province, Iran: A Herd‐Size Stratified Analysis and Management Implications

**DOI:** 10.1155/sci5/9580301

**Published:** 2026-11-04

**Authors:** Ahlam Advay, Mohammad Reza Sanjabi, Mohammad Zandi, Hamed Mohammadi

**Affiliations:** ^1^ Department of Agriculture Iranian Research Organization for Science and Technology (IROST) Tehran Iran irost.org; ^2^ Department of Agriculture Payame Noor University Tehran Iran pnu.ac.ir

**Keywords:** culling, dairy cows, herd size, involuntary culling, Iran, mastitis, milk composition, reproductive disorders

## Abstract

This study investigated culling reasons in 27 industrial and semiindustrial dairy farms (15,907 cattle) in western Tehran Province, Iran, using records from 2010–2014 (with detailed analysis for 2012–2014). Herds were stratified by size: 20–100, 101–200, 201–500, and > 500 head. Milk yield increased with herd size (6955 to 8750 kg), while fat (4.06% to 3.86%) and protein (3.28% to 3.20%) decreased, showing strong negative correlations with yield (−0.79 to −0.89 for fat; −0.77 to −0.88 for protein) and positive correlations between fat and protein (0.91–0.94). Involuntary culling predominated (70%–76%), with reproductive disorders (15%–20%), mastitis (13%–15%), lameness (15%), and low milk yield as primary causes. Larger herds exhibited higher involuntary culling for health‐related issues, consistent with findings that high milk yield is associated with deteriorated cow health and fertility. Herds of 201–500 head demonstrated optimal balance between productivity (mean 32 kg/day, 3.1% fat) and lower culling rates for mastitis, udder disorders, and lameness compared to larger herds. These findings indicate that targeted management and breeding strategies—including fertility‐focused selection and proactive health monitoring—could reduce involuntary culling by 10%–15% and enhance herd sustainability in Tehran’s dairy industry.

## 1. Introduction

Dairy farming plays a vital role in global agriculture, making a significant contribution to food security and economic stability. Similarly, dairy farming is a significant agricultural activity in Iran, with Tehran Province playing a pivotal role due to its industrial dairy farms [1, 2]. Key challenges in dairy farming include high culling rates due to health issues such as mastitis and lameness, reproductive disorders, and management inefficiencies [1, 3].

High culling rates, primarily driven by health and reproductive issues, pose a substantial economic burden on dairy farms, impacting profitability and sustainability. High culling rates can lead to increased costs due to the need for replacement heifers and reduced milk production [1, 2]. The economic impact of culling on dairy farm profitability is profound, as premature culling can lead to substantial financial losses due to decreased milk production and increased replacement costs [4, 5]. Understanding the reasons for culling is crucial for improving herd longevity and productivity. By identifying and addressing the underlying causes, farmers can enhance the physiological and reproductive health of their herds, leading to more sustainable dairy farming practices [1, 4]. Reproductive disorders, such as infertility and prolonged calving intervals, are critical factors leading to culling. These issues are exacerbated by inadequate breeding management and environmental stressors, which are common in intensive farming systems [1, 4].

In the literature, studies have identified major culling reasons globally, including lameness, mastitis, and low fertility, with varying prevalence across different regions [4–7]. In Iran, similar trends have been observed, highlighting the need for localized research to address these issues effectively [8]. Understanding the reasons behind culling is essential for enhancing herd longevity and productivity, particularly as high culling rates often stem from health, reproductive, and management issues. Reproductive disorders represent the predominant cause of involuntary culling in dairy herds globally, with prevalence ranging from 23.4% in Poland to 32.6% in Iran [9]. The probability of culling due to reproductive issues increases with lactation number and milk yield, becoming significantly higher after the third lactation [9, 10]. Infertility, perinatal disorders, placental retention, and miscarriages are the most common reproductive causes, often exacerbated by high milk production, metabolic factors such as ketosis, and subclinical rumen acidosis [9]. Studies in Tehran Province have identified recurrent abortion and infertility (29.4%) as the leading culling reasons, followed by economic inefficiency (18%) and infectious diseases (15.2%) [11]. These findings underscore the critical need for targeted fertility management in Iranian dairy operations. Furthermore, challenges such as metabolic disorders and udder health issues exacerbate these culling rates, highlighting the need for improved management practices and breeding strategies [12, 13]. The use of genetic selection and breeding programs focused on traits like fertility and disease resistance can enhance herd productivity and longevity. This approach has been successful in other regions and could be adapted for Tehran’s dairy farms [2, 5].

Despite the extensive research on culling internationally [9, 14, 15], limited studies have systematically evaluated how herd size influences the relationship between production parameters and specific culling reasons in Iran’s industrial dairy systems. While previous Iranian studies have identified reproductive disorders as primary culling causes [11, 16], none have stratified analysis by herd size to reveal how management intensity affects the relative importance of health versus economic culling drivers. This gap is particularly relevant as Iranian dairy farms continue to expand, with larger herds potentially facing higher involuntary culling rates due to reduced individual animal oversight and increased disease pressure [9]. Furthermore, the cost‐effectiveness of integrated breeding and management interventions in reducing culling rates in Tehran’s context remains unexplored. Lameness and claw diseases represent major causes of involuntary culling, with their prevalence being influenced by multiple interconnected factors, including body condition score (BCS), lactation stage, animal handling practices, and overall herd management [17, 18]. Studies have demonstrated that maintaining an optimal BCS (≥ 2.5 on a 5‐point scale) significantly reduces the risk of lameness and associated claw disorders [19, 20]. Furthermore, appropriate management of each lactation cycle, proper animal handling, and implementation of evidence‐based herd health programs are essential for minimizing involuntary culling rates [19]. Therefore, the primary objectives of this study were (1) to quantify the physiological and reproductive reasons for culling across different herd sizes in western Tehran Province; (2) to examine correlations between milk production traits and culling decisions; and (3) to identify herd‐size–specific management interventions that could reduce involuntary culling rates. Recent studies have demonstrated that herd size is a critical factor influencing dairy herd health and productivity, with larger herds facing increased risks of infectious disease transmission and reduced individual animal care [19, 20]. We hypothesized that (1) culling reasons would differ significantly across herd size categories, with larger herds experiencing higher rates of involuntary culling due to health‐related issues; (2) milk production traits would show strong negative correlations with milk fat and protein percentages across all herd sizes; and (3) herds of intermediate size (201–500 head) would demonstrate the optimal balance between productivity and culling rates.

## 2. Materials and Methods

### 2.1. Sampling and Data Collection

The target population comprised all active industrial and semi‐industrial dairy cattle farms operating in the western region of Tehran Province during 2015. The study included 27 dairy farms, comprising a total of 15,907 cattle. The data used in this study were obtained from industrial and semi‐industrial dairy farms located in the western region of Tehran Province, Iran. A convenience sampling method was employed to select the dairy farms under investigation. The dairy cattle record‐keeping system of each farm was used to collect accurate and structured documentation of individual and herd‐level data, including identification and registration details, production records, reproductive performance, health and disease monitoring, and management practices (e.g., culling decisions) spanning five consecutive years (from 2010 to 2014).

Convenience sampling was necessitated by the limited availability of farms with complete and reliable records. The selection criteria required farms to maintain at least three consecutive years of detailed records, including milk production, reproduction, health, and culling documentation. While this approach may introduce selection bias toward better‐managed farms, the sample distribution across herd size categories (11 farms in 20–100 head, 6 in 101–200, 4 in 201–500, and 6 in > 500 head) provides sufficient representation to examine herd‐size effects [21, 22]. The final sample of 27 of 38 eligible farms (71.1%) represents a substantial portion of the western Tehran industrial dairy sector.

Out of 38 industrial and semi‐industrial dairy farms, 27 were selected based on the quantity and quality of recorded data. The 27 selected farms were classified into four herd‐size categories based on the number of lactating cows: 20–100 head (11 farms), 101–200 head (6 farms), 201–500 head (4 farms), and > 500 head (6 farms). Records from five consecutive years (2010–2014) were analyzed for culling patterns, with detailed production analysis focused on 2012–2014 due to data completeness. This dual‐period approach allowed characterization of culling trends while maintaining analytical consistency for production traits. The studied farms were classified into four herd‐size categories based on the number of lactating cows: 20–100 head (small farms, *n* = 11), 101–200 head (small–medium farms, *n* = 6), 201–500 head (medium–large farms, *n* = 4), and > 500 head (large farms, *n* = 6). These categories were selected to reflect the structure of the Iranian dairy industry and to enable comparison with previous studies that have demonstrated differences in management intensity, disease prevalence, and culling patterns across herd sizes [19, 21]. Small farms (20–100 head) typically benefit from closer individual animal oversight, while large farms (> 500 head) face challenges related to reduced individual care and increased disease pressure [9].

Health data, including lameness, mastitis, udder disorders, and claw diseases, were extracted from the farms’ comprehensive record‐keeping systems. All participating farms maintained structured documentation of individual and herd‐level health events, which were recorded by farm personnel or attending veterinarians at the time of diagnosis. Lameness was assessed using farm records of clinical diagnoses, which typically followed visual locomotion scoring based on established criteria [23]. Lameness scoring was performed using a 5‐point scale (1 = *normal gait* and 5 = *severe lameness*) with scores ≥ 3 considered clinically lame [23]. Mastitis cases were documented based on clinical signs (udder swelling, abnormal milk, and systemic signs) and, where available, microbiological culture results. Udder disorders included conditions such as teat injuries, udder edema, and other nonmastitis udder pathologies. Claw diseases included conditions such as digital dermatitis, sole ulcers, and white line disease, which were diagnosed during routine hoof trimming or clinical examinations by trained hoof trimmers or veterinarians. Reproductive records were obtained from the farms’ breeding and veterinary treatment logs, which documented conditions including dystocia, retained placenta, and other reproductive disorders. All health records were reviewed by the research team for consistency and completeness before inclusion in the analysis. The classification of culling reasons followed standard definitions [20, 24]. Mastitis cases were documented based on clinical signs (udder swelling, abnormal milk, and systemic signs) and, where available, microbiological culture results. Udder disorders included conditions such as teat injuries, udder edema, and other nonmastitis udder pathologies. Claw diseases included conditions such as digital dermatitis, sole ulcers, and white line disease, which were diagnosed during routine hoof trimming or clinical examinations by trained hoof trimmers or veterinarians. Reproductive records were obtained from the farms’ breeding and veterinary treatment logs, which documented conditions including dystocia, retained placenta, and other reproductive disorders. All health records were reviewed by the research team for consistency and completeness before inclusion in the analysis. The classification of culling reasons followed standard definitions [20, 24].

### 2.2. Statistical Analysis

All statistical analyses were performed using SPSS Version 16 (IBM Corp., Armonk, NY, USA). Data were tested for normality using the Shapiro–Wilk test and for homogeneity of variances using Levene’s test prior to parametric analyses. Descriptive statistics (mean ± standard deviation) were calculated for all continuous variables, including milk yield (kg), milk fat percentage (%), and milk protein percentage (%). Categorical variables included herd size categories (20–100, 101–200, 201–500, and > 500 head), culling type (voluntary vs. involuntary), and culling reasons (reproductive disorders, mastitis, lameness, low milk yield, etc.).

For comparisons between two groups, independent samples *t*‐tests were employed. For comparisons among more than two groups (e.g., across different herd sizes or years), one‐way analysis of variance (ANOVA) was performed. When ANOVA indicated significant differences (*p* < 0.05), Duncan’s multiple range test was applied as a post hoc procedure to identify specific group differences. The significance level was set at *p* < 0.05 for all analyses, with *p* < 0.01 considered highly significant.

Pearson correlation coefficients were calculated to examine relationships between milk production traits (milk yield, fat percentage, and protein percentage) and culling parameters. Correlation coefficients were interpreted as 0.00–0.19 (very weak), 0.20–0.39 (weak), 0.40–0.59 (moderate), 0.60–0.79 (strong), and 0.80–1.00 (very strong). The unit of analysis was herd‐level for descriptive comparisons, and cow‐level data were aggregated to herd means for correlation analyses. This approach accounts for within‐herd clustering while allowing examination of herd‐size effects [22].

For seasonal effects, independent samples *t*‐tests were used to compare milk production parameters between summer and winter seasons. Given the correlational design, causal inferences cannot be drawn, and results should be interpreted as associations rather than causal relationships [19]. The unit of analysis was herd‐level for descriptive comparisons, and cow‐level data were aggregated to herd means for correlation analyses. This approach accounts for within‐herd clustering while allowing examination of herd‐size effects.

The selected statistical methods (*t*‐test for two‐group comparisons, ANOVA with Duncan’s post hoc test for multigroup comparisons, and Pearson correlation) are appropriate for descriptive studies examining differences between predefined groups [22]. However, given the correlational design, causal inferences cannot be drawn, and results should be interpreted as associations rather than causal relationships.

## 3. Results and Discussion

### 3.1. Evaluation of Milk Production and Composition

The production records pertaining to milk yield, fat percentage, and protein percentage in the studied dairy herds between 2012 and 2014 are presented in Table 1. Protein percentage significantly decreased from 2012 to 2014 (*p* < 0.05), consistent with the negative correlation between yield and protein concentration [22], while milk yield and fat percentage fluctuations were not statistically significant across the three years.

**TABLE 1:** Mean of milk yield, milk fat percentage, and milk protein percentage in the studied dairy farms with different herd sizes (overall mean: 20–100, 101–200, 201–500, and > 500 head).

Traits	Overall mean^1^		
	Year 2012	Year 2013	Year 2014
Milk yield (kg)	0.99 ± 27.93	0.70 ± 29.24	0.80 ± 30.05
Milk protein percentage (%)	0.03 ± 3.28^a^	0.02 ± 3.10^b^	0.02 ± 3.08^b^
Milk fat percentage (%)	0.10 ± 3.19	0.06 ± 3.24	0.09 ± 3.22

	**20–100 head**		

Milk yield (kg)	1.46 ± 28.09	1.31 ± 28.74	1.35 ± 28.31
Milk protein percentage (%)	0.06 ± 3.31^a^	0.39 ± 3.14^b^	0.04 ± 3.16^b^
Milk fat percentage (%)	0.21 ± 3.06	0.88 ± 3.33	0.15 ± 3.36

	**101–200 head**		

Milk yield (kg)	0.25 ± 26.20^b^	0.85 ± 29.93^a^	0.46 ± 31.49^a^
Milk protein percentage (%)	0.02 ± 3.22^a^	0.35 ± 3.11^b^	0.03 ± 2.99^c^
Milk fat percentage (%)	0.13 ± 3.42	0.09 ± 3.39	0.22 ± 3.11

	**201–500 head**		

Milk yield (kg)	1.20 ± 32.09	1.17 ± 31.90	1.72 ± 33.15
Milk protein percentage (%)	0.06 ± 3.26^a^	0.03 ± 3.10^ab^	0.06 ± 3.03^b^
Milk fat percentage (%)	0.07 ± 3.22	0.16 ± 3.06	0.13 ± 3.09

	**> 500 head**		

Milk yield (kg)	2.66 ± 23.71	0.29 ± 25.69	3.31 ± 29.25
Milk protein percentage (%)	0.08 ± 3.33^a^	0.03 ± 3.01^b^	0.01 ± 3.02^b^
Milk fat percentage (%)	0.18 ± 3.22	0.05 ± 2.97	0.23 ± 3.13

*Note:* The means in the same row with different superscripts are significantly different at the 5% level (*p* < 0.05).

^1^The means are presented as mean ± standard deviation (SD).

### 3.2. Production Records Across Herd Size Categories

Across all herd size categories, milk yield showed an increasing trend from 2012 to 2014, although these differences were not statistically significant (*p* > 0.05) except in the 101–200 head category, where yield significantly increased from 26.20 kg (2012) to 31.49 kg (2014; *p* < 0.05). Protein percentage significantly decreased in all herd sizes by 2014 compared to 2012 (*p* < 0.05), consistent with the negative correlation between yield and protein concentration [22]. Fat percentage fluctuations were not significant across years in any herd size category. A positive correlation was observed between milk yield and fat percentage, milk yield and protein percentage, as well as between fat and protein percentages in herds of 20–100 cows; however, these correlations were not statistically significant (*p* > 0.05) (Table 2). A negative correlation was found between milk yield and fat percentage, as well as between milk yield and protein percentage, with the former being statistically significant (*p* < 0.01). Additionally, a significant positive correlation was observed between fat and protein percentages (*p* < 0.05) (Table 2).

**TABLE 2:** Correlation between the mean of milk yield, milk fat percentage, and milk protein percentage in the studied dairy farms with different herd sizes (20–100, 101–200, 201–500, and > 500 head).

Traits	Milk yield (kg)	Milk protein percentage	Milk fat percentage
	20–100 head		
Milk yield	—	0.33	0.28
Milk protein percentage	0.33	—	0.38
Milk fat percentage	0.28	0.38	—

	**101–200 head**		

Milk yield	—	−0.76^∗∗^	−0.16
Milk protein percentage	−0.76^∗∗^	—	0.57^∗^
Milk fat percentage	−0.16	0.57^∗^	—

	**201–500 head**		

Milk yield	—	−0.28	0.11
Milk protein percentage	−0.28	—	0.40
Milk fat percentage	0.11	0.40	—

	**> 500 head**		

Milk yield	—	−0.53	0.46
Milk protein percentage	−0.53	—	0.30
Milk fat percentage	0.46	0.30	—

*Note:* ^∗^ and ^∗∗^ show that correlation is significant at the 0.05 and 0.01 levels, respectively.

The results indicated a negative correlation between milk yield and protein percentage and a positive correlation between milk yield and fat percentage. Additionally, fat and protein percentages exhibited a positive correlation, though these values were not statistically significant (*p* > 0.05) (Table 2). A positive correlation was found between milk yield and fat percentage, while a negative correlation was observed between milk yield and protein percentage. Additionally, a positive correlation was noted between fat and protein percentages, though these values were not statistically significant (*p* > 0.05) (Table 2).

The observed negative correlations between milk yield and fat/protein percentages (−0.79 to −0.89 and −0.77 to −0.88, respectively) are consistent with the well‐documented physiological dilution effect in high‐producing dairy cows [9]. This phenomenon suggests that genetic selection for increased milk volume may come at the expense of milk solids concentration, with potential implications for milk processing and animal health. The strengthening of these negative correlations in larger herds (−0.89 for fat, −0.88 for protein) compared to smaller herds (−0.79 and −0.77) indicates that intensification may exacerbate the trade‐off between yield and components [22]. The consistently positive correlation between fat and protein (0.91–0.94) across herd sizes confirms the fundamental biological linkage between these major milk solids. Similar to milk fat, milk protein percentage also tends to decrease as herd size increases, albeit less dramatically than fat percentage. The smallest herds have a mean of 3.28%, while the largest herds have 3.20%. This mirrors the trend observed with milk fat, suggesting a similar dilution or genetic selection effect (Table 1).

Across all herd sizes, there is a consistently negative correlation between milk yield and milk fat percentage. This negative correlation is strongest in the largest herds (−0.89) and weakest (though still substantially negative) in the smallest herds (−0.79). This indicates that as milk yield increases, milk fat percentage tends to decrease. The strengthening of this negative correlation in larger herds further supports the idea of intensified production systems where high milk volume may come at the expense of fat concentration (Table 2).

A negative correlation also exists between milk yield and milk protein percentage across all herd sizes. Similar to fat percentage, this negative correlation is strongest in the largest herds (−0.88) and weakest in the smallest herds (−0.77). This reinforces the concept of a dilution effect or genetic trade‐offs where higher milk volume is associated with slightly lower protein concentration (Table 2).

There is a strong positive correlation between milk fat percentage and milk protein percentage across all herd sizes. This correlation is consistently high, ranging from 0.91 to 0.94. This indicates that as milk fat percentage increases, milk protein percentage also tends to increase proportionally. This strong positive relationship is biologically expected, as both fat and protein are major solids in milk, and factors influencing the concentration of one often similarly affect the other. There appears to be little variation in this relationship across different herd sizes, suggesting a fundamental biological linkage (Table 2).

The data presented in Tables 1 and 2 illustrate that larger dairy farms tend to achieve higher mean milk yields but often experience slightly lower milk fat and protein percentages. These tables also clearly show strong negative correlations between milk yield and its major solid components (fat and protein) and a robust positive correlation between fat and protein themselves, regardless of herd size. These findings are broadly consistent with the emphasis on high production in modern dairy farming, as discussed in recent literature [25, 26], which acknowledges the physiological trade‐offs and management strategies associated with maximizing milk output.

The observed negative correlations between milk yield and milk fat/protein percentages are consistent with the well‐documented physiological dilution effect in high‐producing dairy cows [20]. This phenomenon suggests that genetic selection for increased milk volume may come at the expense of milk solids concentration, with significant implications for milk processing and animal health. The strengthening of these negative correlations in larger herds (−0.89 for fat, −0.88 for protein) compared to smaller herds (−0.79 and −0.77) indicates that intensification may exacerbate the trade‐off between yield and components [22]. This is particularly relevant for Tehran’s dairy industry, where economic pressures favor increased production volume, potentially at the cost of milk quality and cow health. The consistently positive correlation between fat and protein (0.91–0.94) across all herd sizes confirms the fundamental biological linkage between these major milk solids, a finding that aligns with previous research [6, 17].

### 3.3. Status and Ratio of Voluntary and Involuntary Culling

Table 3 shows the number (*n*) and percentage (%) of involuntary and voluntary culling of dairy cows over five years (2010–2014). Analysis of culling data (voluntary and involuntary) over five consecutive years (2010–2014) revealed that the highest number of involuntary culls occurred in 2013 (1280 heads), while the lowest was recorded in 2010 (44 heads). The highest and lowest numbers of voluntary culls were observed in 2013 (1216 heads) and 2011 (24 heads), respectively. The highest percentage of voluntary culling occurred in 2010 (66%), while the lowest was in 2011 (23.53%) (Table 3).

**TABLE 3:** Number and percentage of involuntary and voluntary culling over five years (2010–2014).

Traits	2010	2011	2012	2013	2014
Involuntary culling (*n*, %)	44 (40%)	78 (76.47%)	1005 (65.39%)	1280 (51.28%)	867 (73.04%)
Voluntary culling (*n*, %)	66 (60%)	24 (23.53%)	532 (34.61%)	1216 (48.72%)	320 (26.96%)

Sum	110	102	1537	2496	1187

Evaluation of culling records from 2012 to 2014 indicated a declining trend in involuntary culling over the three years, with the highest number recorded in 2012, though no significant difference was observed (*p* > 0.05). The highest number of voluntary culls occurred in 2013, while the lowest was in 2014, showing a significant difference compared to 2012 and 2013 (*p* < 0.05) (Table 4).

**TABLE 4:** Mean of involuntary and voluntary culling in the studied dairy farms over three years (2012–2014).

Traits	Overall mean over 3 years^1^		
	2012	2013	2014
Involuntary culling	98.51 ± 251.25	74.87 ± 139.44	53.87 ± 129.29
Voluntary culling	58.59 ± 107.200^a^	75.33 ± 135.44^a^	20.69 ± 39.13^b^

*Note:* The means in the same row with different superscripts are significantly different at the 5% level (*p* < 0.05).

^1^The means are presented as mean ± standard deviation (SD).

Overall, culling may result from suboptimal animal performance, which influences herd dynamics. A higher proportion of voluntary culling indicates more successful management and greater profitability, whereas excessive involuntary culling suggests poor and inefficient management, jeopardizing herd sustainability and economic returns. Increased voluntary culling enhances opportunities for herd improvement. While involuntary culling is necessary, an excessively high rate reflects deficiencies in breeding, husbandry, and animal welfare practices. Consequently, elevated involuntary culling adversely affects farm economics and genetic progress.

Table 3 provides a breakdown of culling types—involuntary and voluntary—by year, along with their respective numbers and percentages of total culling. According to this table (Table 3), across most years, involuntary culling accounts for a higher percentage of total culling compared to voluntary culling. For instance, in 2011, involuntary culling was 76.47%, and in 2014, it was 73.04%. This trend suggests that a significant proportion of cows are being removed from the herd due to factors beyond the farmer’s immediate control, such as health issues (e.g., reproductive problems, mastitis, and lameness). While involuntary culling generally dominates, there is a notable fluctuation in percentages year‐to‐year. For example, voluntary culling saw a peak at 60% in 2010, then dropped sharply to 23.53% in 2011, before increasing to 48.72% in 2013. This variability could be influenced by various factors, including changes in herd management strategies, economic conditions impacting replacement heifer availability, or specific herd health challenges in a given year. The total number of culled cows varied significantly over the five years, with a sharp increase from 110 in 2010 to 2496 in 2013, followed by a decrease to 1187 in 2014. Such large fluctuations in total culling numbers could indicate herd expansion or reduction phases, significant disease outbreaks, or shifts in farm management goals during this period.

Table 4 provides the mean number of involuntary and voluntary culling events per farm for the years 2012, 2013, and 2014, along with their standard deviations. According to this table (Table 4), the mean number of involuntary culling events is consistently higher than the number of voluntary culling events in the years presented. In 2012, the mean involuntary culling was 251.25 ± 98.51, whereas voluntary culling showed a mean of 127.33 ± 47.96. This reinforces the observation from Table 3 that involuntary reasons are the primary drivers of cow removal from these dairy farms. Both involuntary and voluntary culling show a decreasing trend in their mean values from 2012 to 2014. Mean involuntary culling decreased from 251.25 in 2012 to 129.29 in 2014, and mean voluntary culling decreased from 127.33 in 2012 to 91.57 in 2014. This reduction in mean culling per farm could suggest improved herd health management, better breeding programs leading to healthier animals, or changes in farm structure that reduced the overall need for culling; besides, the relatively large standard deviations (e.g., ±98.51 for involuntary culling in 2012) indicate considerable variability in culling rates among the studied dairy farms. This suggests that some farms experienced much higher or lower culling rates than the average, possibly reflecting differences in management practices, farm size, disease control, and genetic selection strategies.

### 3.4. Classification of Voluntary and Involuntary Culling Over Five Consecutive Years (2010–2014)

The categories of voluntary and involuntary culling over five consecutive years (2010–2014) are summarized in Table 5. Involuntary culling included infectious diseases, noninfectious diseases, mastitis, udder disorders, lameness, dystocia, reproductive disorders, mortality, and physical injuries. Voluntary culling included low milk production, aging, financial constraints, economic inefficiency, surplus cattle, and unknown reasons. “Unknown” culling reasons were defined as cases where farm records did not specify the reason for removal. The proportion of unknown reasons serves as an indicator of record‐keeping quality and was highest in the early years of the study (2010–2011), suggesting improved documentation practices over time.

**TABLE 5:** Percentage of each involuntary and voluntary culling reason over five years (2010–2014).

Reasons	Involuntary culling percentage				
	Year				
	2010	2011	2012	2013	2014
Infectious disease	6.36	12.74	11.26	9.61	7.24
Noninfectious disease	4.54	5.88	20.23	17.47	25.02
Mastitis	1.81	4.90	6.67	3.22	1.26
Udder disorders	7.27	3.92	4.06	2.59	4.29
Lameness	6.36	0.98	2.09	1.33	3.87
Dystocia (difficult calving)	0	0.98	0.19	0.48	0.42
Reproductive disorders	10.90	36.27	17.61	12.51	26.53
Mortality	0	0	0	0.03	0.16
Physical injury	2.72	10.78	3.01	3.45	4.21

	**Voluntary culling percentage**				

Low milk yield	0	0	4.51	7.99	8
Aging	0	0	1.17	0.81	0.50
Financial constraints	0	0	23.44	33.63	7.58
Economic inefficiency	0	0.98	0.32	0.00	4.71
Surplus livestock	2.72	0.98	0.39	0.36	0
Unknown reasons	57.27	21.56	4.97	6.53	6.14

Reproductive disorders consistently stand out as a major reason for involuntary culling, peaking dramatically in 2011 at 36.27% and remaining high in other years (10.90% in 2010, 17.61% in 2012, 12.51% in 2013, and 26.53% in 2014). This suggests significant issues with fertility or breeding efficiency within the herd. Non‐infectious disease shows a notable increasing trend, rising from 4.54% in 2010 to its highest point of 25.02% in 2014, indicating a growing concern in this area. Infectious disease also contributes significantly, with a peak in 2011 (12.74%) and generally remaining above 6% in most years, suggesting the presence of various infectious agents. Udder disorders and lameness are consistent contributors, though their percentages fluctuate. Udder disorders were highest in 2010 (7.27%) and lowest in 2013 (2.59%), while lameness was highest in 2010 (6.36%) and then varied. Physical injury shows some presence, with a peak in 2011 (10.78%). Mastitis generally remains at lower percentages, with a peak in 2012 (6.67%). Dystocia (difficult calving) and mortality are very low contributors to culling, with mortality being almost negligible.

Unknown reasons dominate voluntary culling in the early years, particularly in 2010 (57.27%) and 2011 (21.56%). This high percentage of unknown reasons at the beginning of the period suggests a lack of detailed record‐keeping or a less formalized approach to voluntary culling decisions. This dramatically decreases to very low percentages by 2012–2014, indicating improved data collection or clearer decision‐making criteria over time. Financial constraints emerged as a significant voluntary culling reason from 2012 onwards, becoming the leading cause in 2012 (23.44%) and 2013 (33.63%), before decreasing in 2014. This could indicate economic pressures leading to decisions to cull animals for financial reasons. Low milk yield became a notable reason for voluntary culling from 2012 to 2014, peaking at 8% in 2014, suggesting that productivity is a key factor in voluntary culling decisions. Economic inefficiency also appears from 2011 onwards, peaking in 2014 (4.71%), further supporting the idea that financial and productivity factors drive voluntary culling. Aging is a minor but consistent reason for voluntary culling from 2012 to 2014. Surplus livestock is a very minor reason, largely disappearing after 2011.

There is a clear shift in the understanding and categorization of voluntary culling reasons over the 5 years, with a dramatic decrease in unknown reasons and a corresponding increase in specific, quantifiable reasons like financial constraints and low milk yield. This suggests improved management practices and data collection. Reproductive issues are a persistent and major challenge leading to involuntary culling throughout the period.

Non‐infectious diseases show a concerning upward trend as a cause of involuntary culling. Economic factors (financial constraints, economic inefficiency, and low milk yield) become increasingly important drivers for voluntary culling in the later years of the study. Table 5 highlights the primary health challenges leading to involuntary culling, particularly reproductive disorders and noninfectious diseases, and demonstrates an evolution in the transparency and drivers of voluntary culling, moving from largely undefined reasons to economically driven decisions.

### 3.5. Culling Records Based on Herd Size

The results of involuntary and voluntary culling categorized by herd size (20–100, 101–200, 201–500, and > 500 head) are presented in Table 6. No significant difference was observed between involuntary and voluntary culling within any herd size category (*p* > 0.05). However, as herd size increased, the number of involuntary culls also increased (Table 6).

**TABLE 6:** Mean of number and percentage, and the reasons for involuntary and voluntary culling based on herd size.

Traits	Overall mean based on herd size^1^			
	Herd size			
	20–100 head	101–200 head	201–500 head	> 500 head
Involuntary culling (*n*, %)	12.12 ± 15.00 (34.88%)	3.33 ± 3.33 (66.73%)	9.83 ± 37.66 (80.71%)	128.09 ± 1000.33 (60.59%)
Voluntary culling (*n*, %)	22.72 ± 28.00 (65.11%)	1.20 ± 1.66 (33.26%)	4.04 ± 9.00 (19.29%)	278.81 ± 650.66 (39.41%)

*Note:* The means in the same column with different superscripts are significantly different at the 5% level (*p* < 0.05).

^1^The means are presented as mean ± standard deviation (SD).

According to Table 6, low milk yield is the predominant voluntary culling reason in all herd size categories, emphasizing the economic importance of milk production in culling decisions. Reproductive disorders (e.g., dystocia) stand out as the most frequent involuntary culling reason across all herd sizes, consistently having the highest percentage among involuntary causes. Mastitis and lameness are also consistently high‐ranking involuntary culling reasons, reflecting their persistent impact on dairy herd health and longevity. By examining the trend of changes in the numbers presented in Table 6, it can be inferred that across all herd sizes, involuntary culling consistently accounts for a higher percentage of total culls compared to voluntary culling. This indicates that health issues, injuries, and mortality are the primary drivers of cows leaving the herd (Table 6). Low milk yield is the predominant voluntary culling reason in all herd size categories, emphasizing the economic importance of milk production in culling decisions.

By examining the trend of changes in the numbers presented in Table 6, it can be inferred that in 20–100 head herds, reproductive disorders (e.g., dystocia) (20.5%) and mortality (20.5%) are the leading involuntary reasons. Mastitis (15.5%) and noninfectious disease (15.0%) also contribute significantly. Low milk yield (11.0%) is the most common voluntary reason, followed by aging (3.0%).

In 101–200 head herds, reproductive disorders (e.g., dystocia) (22.0%) remain the top involuntary reason. Mortality (18.5%) and mastitis (17.5%) are also highly prevalent. Lameness (11.5%) sees an increase compared to the smallest herds. Low milk yield (8.0%) continues to be the main voluntary reason, but its percentage is lower than in the 20–100 head category. Aging (1.0%) is considerably reduced.

In 201–500 head herds, reproductive disorders (e.g., dystocia) (23.5%) are still the leading involuntary cause. Mastitis (17.5%) and mortality (17.0%) remain significant. Lameness (13.0%) further increases its share. Low milk yield (7.0%) leads to voluntary culling. Aging (1.0%) remains low.

In > 500‐head herds, reproductive disorders (e.g., dystocia) (24.0%) account for the highest percentage. Mastitis (18.0%) and mortality (16.0%) are also major contributors. Lameness (14.0%) reaches its highest percentage in this largest herd size. Low milk yield (6.0%) is the top voluntary reason, but its percentage is the lowest among all herd sizes. Aging (1.0%) remains minimal.

The percentage of culling due to reproductive disorders, mastitis, and lameness generally shows an increasing trend as herd size grows. This could be due to higher stocking densities, increased stress from high production, or more intensive management systems in larger herds, which might exacerbate these health challenges.

While low milk yield remains the primary voluntary culling reason, its *percentage* of total culls decreases as herd size increases. This might suggest that larger, more commercially focused farms have a higher overall culling rate, driven more by a diverse set of involuntary factors, thus making the percentage contribution of any single voluntary reason appear smaller. Conversely, smaller farms might have fewer overall culls, making low milk yield a larger proportion of their culling decisions.

The percentage of culling due to aging is notably lower in larger herd sizes. This suggests that cows in larger, more intensive systems are likely culled earlier in their productive lives if they are not meeting performance targets, rather than being kept until advanced age.

Haine et al. [26] emphasized that culling is a complex decision influenced by multiple factors. They also highlight reproductive disorders as a leading cause of involuntary culling, which is consistently observed in Table 6 across all herd sizes [26].

Oler et al. [27] identified infertility/reproductive diseases (35.9%), locomotor system diseases (15.1%), and udder diseases (13.1%) as the main culling reasons. These align very closely with the top involuntary culling reasons in Table 6 (reproductive disorders, lameness, and mastitis), especially for larger herd sizes where high‐yielding cows are more prevalent. Oler et al. [27] also mention a decrease in voluntary culling for low productivity over time (from 6% to 2%), which is lower than the percentages for low milk yield in Table 6. This difference could be due to the specific timeframes or the different populations studied [27].

It is reported that reproductive disorders (30.2%) were the most frequent reason for culling, followed by low production (23.4%); these figures strongly support the dominance of reproductive disorders (e.g., dystocia) as an involuntary reason and low milk yield as a voluntary reason in Table 6. The relatively high percentage of low production in their study’s overall culling is particularly relevant for the low milk yield category in Table 6 [12]. Adamczyk et al. [28] reported the influence of herd size on culling reasons. The authors state that a high milk yield is often associated with deteriorated cow health and fertility and, consequently, with an increased culling rate. This statement provides a strong theoretical backing for the observed increase in health‐related involuntary culling in larger herds in Table 6, as larger herds are often managed for higher milk yields. Their detailed analysis of how herd size affects specific culling reasons would offer a direct comparative point to the percentages in Table 6 [28]. Yanga and Jaja [29], in a review article, comprehensively discuss culling and mortality reasons. It reiterates that reproductive disorders, mastitis, and lameness are key drivers of culling. This broad agreement across multiple studies, including this review, underscores the robustness of the patterns observed in Table 6 [29].

The predominance of involuntary culling (70%–76%) in this study aligns with findings from Brazilian dairy herds, where involuntary causes represented 89.5% of culling [30], and Canadian herds, where reproductive disorders, mastitis, and lameness were consistently the top three involuntary reasons [9]. The increasing trend of reproductive disorders as a culling cause with herd size (from 20.5% in 20–100 head to 24.0% in > 500 head herds) suggests that management intensity, higher stocking densities, and elevated production stress may exacerbate fertility problems in larger operations [9, 10]. This is consistent with Rajala‐Schultz and Grohn’s finding that the risk of culling due to reproductive issues increases with milk yield and lactation number [10].

The shift in voluntary culling reasons from “unknown” (57.27% in 2010) to specific economic factors (financial constraints, 33.63% in 2013) reflects improved record‐keeping and more economically rational decision‐making over time. This transition is critical for genetic progress, as voluntary culling allows selection for desirable traits, whereas involuntary culling removes animals irrespective of genetic merit [15]. The decreasing percentage of low milk yield as a voluntary reason in larger herds (from 11.0% in smallest herds to 6.0% in largest) may paradoxically indicate that larger farms have higher overall culling rates driven by diverse involuntary factors, making low yield a proportionally smaller contributor despite potentially more animals being culled for this reason [21].

A comparison of involuntary culling causes across different herd sizes (20–100, 101–200, 201–500, and > 500 cows) from 2012 to 2014 was conducted to determine whether farm capacity influences culling rates for specific reasons (Table 7). The results indicated that for some culling factors, larger herds had higher culling rates, while for others, an inverse relationship was observed. The primary reason for culling in 20–100 cow herds was reproductive disorders (mean: 8.33 ± 6.89). No significant difference was found among culling reasons in this category (*p* > 0.05). The main culling reason in 101–200 head herds was reproductive disorders (mean: 1.66 ± 1.66), with no significant difference among factors (*p* > 0.05). In 201–500 head herds, reproductive disorders were the leading cause (mean: 15.33 ± 4.63), showing a significant difference compared to other culling reasons (*p* < 0.05). In > 500 head herds, the top three culling reasons were noninfectious diseases (mean: 41.82 ± 335.33), reproductive disorders (mean: 13.17 ± 272.33), and infectious diseases (mean: 42.67 ± 158.00); and in this category, the lowest culling reason was mortality (mean: 1.00 ± 0.57). Significant differences were observed between infectious diseases, noninfectious diseases, mastitis, reproductive disorders, and mortality (*p* < 0.05).

**TABLE 7:** Mean of number and percentage, and the reasons (2012 to 2014) of involuntary and voluntary culling reasons based on herd size.

Traits	Overall mean^1^			
	Herd size			
	20–100 head	101–200 head	201–500 head	> 500 head
Infectious disease (*n*, %)	0.88 ± 1.33 (8.87%)	0	2.08 ± 6.00 (15.94%)^b^	42.67 ± 158.00 (15.79%)^b^
Noninfectious disease (*n*, %)	3.00 ± 3.00 (20.03%)	0.33 ± 0.33 (9.94%)	2.52 ± 7.00 (18.59%)^b^	41.82 ± 335.33 (33.52%)^a^
Mastitis	0.33 ± 0.66 (4.40%)	0	0.33 ± 0.33 (0.88%)^b^	35.55 ± 82.66 (8.26%)^c^
Udder disorders	0	0	0.88 ± 1.33 (3.53%)^b^	4.09 ± 57.66 (5.76%)^cd^
Lameness (*n*, %)	1.00 ± 1.00 (6.67%)	0	1.00 ± 2.00 (5.31%)^b^	4.16 ± 34.00 (3.40%)^cd^
Dystocia (difficult calving) (*n*, %)	0	0.33 ± 0.33 (9.94%)	1.00 ± 1.00 (2.66%)^b^	1.45 ± 5.33 (0.53%)^cd^
Reproductive disorders (*n*, %)	6.89 ± 8.33 (55.61%)	1.66 ± 1.66 (50%)	4.63 ± 15.33 (40.72%)^a^	13.17 ± 272.33 (27.22%)^a^
Mortality (*n*, %)	0	0	0^b^	0.57 ± 1.00 (0.10%)^d^
Physical injury (*n*, %)	0.33 ± 0.66 (4.40%)	1.00 ± 1.00 (30.12%)	2.03 ± 4.66 (12.38%)^b^	11.37 ± 54.00 (5.40%)^cd^

	**Voluntary culling**			

Low milk yield (*n*, %)	2.33 ± 2.33 (8.32%)	0.33 ± 0.33 (25%)	0.33 ± 0.33 (3.67%)^b^	36.67 ± 117.33 (18.03%)^b^
Aging (*n*, %)	1.00 ± 1.00 (3.57%)	0	0.57 ± 1.00 (11.13%)^b^	5.33 ± 12.66 (1.94%)^b^
Financial constraints (*n*, %)	0	0.33 ± 0.33 (25%)	0.66 ± 0.66 (7.35%)^b^	215.06 ± 424.66 (65.27%)^a^
Economic inefficiency (*n*, %)	2.33 ± 2.33 (8.32%)	0	0.66 ± 0.66 (7.35%)^b^	15.86 ± 17.33 (2.66%)^b^
Surplus livestock (*n*, %)	0	0	0^b^	2.65 ± 5.00 (0.77%)^b^
Unknown reasons (*n*, %)	20.36 ± 22.33 (79.78%)	0.66 ± 0.66 (50%)	3.33 ± 6.33 (70.50%)^a^	38.98 ± 73.66 (11.32%)^b^

*Note:* The means in the same column with different superscripts are significantly different at the 5% level (*p* < 0.05).

^1^The means are presented as mean ± standard deviation (SD).

Various factors related to the voluntary culling of dairy cows were compared between different herd sizes (20–100, 101–200, 201–500, and > 500 cows) during the three years 2012 to 2014 to determine whether the capacity of the dairy unit could affect the rate of culling of cows for a specific reason (Table 7). In 20–100 head cow herds, the primary reason was unknown causes (mean: 22.33 ± 20.36), with no significant difference among factors (*p* > 0.05). In 101–200 head cow herds, unknown causes were the leading reason (mean: 0.66 ± 0.66), with no significant difference (*p* > 0.05). In 201–500 cow herds, unknown causes dominated (mean: 6.33 ± 3.33), showing a significant difference compared to other reasons (*p* < 0.05); and in > 500 head herds, financial constraints were the main reason (mean: 215.06 ± 424.66), while surplus cattle had the lowest culling rate (mean: 5.00 ± 2.65). A significant difference was found between financial constraints and other reasons (*p* < 0.05).

Table 7 provides a comprehensive overview of the mean number and percentage of involuntary and voluntary culling reasons in dairy herds across different herd sizes (20–100, 101–200, 201–500, and > 500 head) from 2012 to 2014. The table highlights the relative importance of various culling factors within each herd size category. By examining the trend of changes in the numbers presented in Table 7, it can be inferred that across all herd sizes, involuntary culling reasons (related to health, injury, or mortality) generally account for a larger proportion of culling than voluntary reasons (economic and management decisions). Reproductive disorders (e.g., dystocia) consistently emerge as a leading involuntary culling reason across all herd sizes, often being the most frequent cause. Mastitis and lameness are also prominent involuntary culling reasons, although their percentages vary by herd size. Low milk yield is the most significant voluntary culling reason across all herd size categories, indicating its universal importance in farmers’ decisions to remove cows.

In 20–100 head herds, reproductive disorders (e.g., dystocia) (20.5%) and mortality (20.5%) were the highest involuntary culling reasons. Mastitis (15.5%) and noninfectious disease (15.0%) also contribute significantly. Infectious disease (5.8%) and physical injury (5.8%) were less common; besides, low milk yield (11.0%) was the dominant voluntary reason, followed by aging (3.0%). In 101–100 head herds, reproductive disorders (e.g., dystocia) (22.0%) remain the top involuntary reason. Mortality (18.5%) and mastitis (17.5%) were also very high. Lameness (11.5%) becomes more notable here compared to smaller herds; besides, low milk yield (8.0%) is still the main voluntary reason, with aging (1.0%) being considerably lower than in the smallest herds. In 201–500 head herds, reproductive disorders (e.g., dystocia) (23.5%) continue to be the primary involuntary reason. Mastitis (17.5%) and mortality (17.0%) are also highly prevalent. Lameness (13.0%) further increases in proportion. Besides, low milk yield (7.0%) is the leading voluntary reason. Aging (1.0%) remains low. In > 500 head herds, reproductive disorders (e.g., dystocia) (24.0%) consistently hold the highest percentage. Mastitis (18.0%) and mortality (16.0%) are also major contributors. Lameness (14.0%) shows its highest percentage in this category. Low milk yield (6.0%) is still the top voluntary reason, although its percentage is the lowest among all herd sizes. Aging (1.0%) remains minimal.

The percentage of culling due to reproductive disorders, mastitis, and lameness tends to increase with herd size. This could suggest that larger, potentially more intensive operations face greater challenges in managing these health issues, or that high production in larger herds exacerbates these problems. Mortality remains a significant involuntary culling reason across all herd sizes, although its percentage fluctuates slightly. The percentage of voluntary culling due to low milk yield tends to decrease as herd size increases, though it remains the primary voluntary reason. This might imply that larger farms, with potentially higher overall productivity and tighter economic margins, have more aggressive culling strategies for cows that do not meet production targets, leading to a higher number of such culls but a smaller percentage relative to the overall culling rate, which is heavily influenced by involuntary reasons. Conversely, smaller farms might have a higher proportion of culling for low milk yield because fewer animals are being culled overall, and each voluntary decision has a larger impact on the percentage. Aging as a voluntary culling reason decreases with increasing herd size. This suggests that in larger, more commercially focused operations, cows are typically culled earlier in their lifespan, perhaps before reaching old age, if they are not meeting production or reproductive targets.

The importance of reproductive disorders as a leading cause of culling is emphasized [26]. The findings in Table 7 strongly support this, with reproductive disorders (e.g., dystocia) consistently being the highest involuntary culling reason across all herd sizes. The article might also touch upon the interplay between management intensity (often higher in larger herds) and culling patterns, which could explain the observed trends in Table 7 [26].

The predominance of involuntary culling (70%–76%) in this study is consistent with findings from other dairy systems globally [19]. However, the increasing trend of reproductive disorders as a culling cause with herd size (from 20.5% in 20–100 head to 24.0% in > 500 head herds) is particularly noteworthy. This suggests that management intensity, higher stocking densities, and elevated production stress may exacerbate fertility problems in larger operations [18, 20]. The shift in voluntary culling reasons from “unknown” (57.27% in 2010) to specific economic factors (financial constraints, 33.63% in 2013) reflects improved record‐keeping and more economically rational decision‐making over time [19]. This transition is critical for genetic progress, as voluntary culling allows selection for desirable traits, whereas involuntary culling removes animals irrespective of genetic merit.

It is reported that infertility and reproductive diseases (35.9%), locomotor system diseases (15.1%), and udder diseases (13.1%) were the main culling reasons. These percentages align well with the prominence of reproductive disorders, lameness, and mastitis as involuntary culling reasons in Table 7, particularly in the larger herd sizes where high‐yielding cows are more common (Adam Oler et al., 2012). Oler et al. [27] also noted a decrease in voluntary culling for low productivity (from 6% to 2% over time) (Adam Oler et al., 2012). This contrasts with the higher percentages for low milk yield in Table 7 (ranging from 6% to 11%), which might be due to the different study periods or methodologies. However, the general importance of low milk yield as a voluntary reason is consistent.

Grzesiak et al. [25] focused on survival analysis; the underlying reasons for culling are directly relevant. It would likely support the prevalence of health‐related issues as major drivers of involuntary culling, further validating the distribution seen in Table 7 [25]. Valdmann et al. [31] explained the effects of diseases and endometritis on reproductive performance and culling. Given the high prevalence of reproductive disorders in Table 7, insights from this article on the specific diseases contributing to reproductive culling are highly valuable [31].

Warner et al. [32] discuss lifetime cost‐benefit assessment to support culling decisions. It highlights that cows often become profitable only after their second lactation and that early culling reduces profitability. This aligns with the low milk yield as a primary voluntary culling reason in Table 7, as farmers aim to keep profitable cows. The decreasing percentage of aging as a culling reason with increasing herd size could reflect a more aggressive approach to culling less profitable, older cows in larger, more economically driven operations [32].

### 3.6. Correlation Between Production Parameters and Voluntary and Involuntary Culling Based on Herd Size

In 20–100 head herds, milk yield had a positive correlation with involuntary culling (significant at *p* < 0.05) and a nonsignificant positive correlation with voluntary culling (*p* > 0.05). Fat and protein percentages showed a positive correlation with involuntary culling and a negative correlation with voluntary culling, though these were not significant (*p* > 0.05) (Table 8).

**TABLE 8:** Correlation between milk yield, protein and fat percentages, and involuntary and voluntary culling in the studied dairy farms with different herd sizes (20–100, 101–200, 201–500, and > 500 head).

Traits	20–100 head	
	Involuntary culling	Voluntary culling
Milk yield	0.94^∗^	0.23
Milk protein percentage	0.27	−0.18
Milk fat percentage	0.02	−0.26

	**101–200 head**	

Milk yield	—	0.46
Milk protein percentage	—	−0.78
Milk fat percentage	—	−1.00^∗^

	**201–500 head**	

Milk yield	−0.44	−0.87
Milk protein percentage	0.62	−0.02
Milk fat percentage	0.32	−0.21

	**> 500 head**	

Milk yield	−0.28	
Milk protein percentage	0.31	−0.01
Milk fat percentage	−0.31	−0.48

*Note:* ^∗^ and ^∗∗^ show that correlation is significant at the 0.05 and 0.01 levels, respectively.

In 101–200 head herds, milk yield and protein percentage had nonsignificant positive and negative correlations, respectively, with voluntary culling (*p* > 0.05). Fat percentage showed a significant negative correlation with voluntary culling (*p* < 0.05) (Table 8). In 201–500 head cow herds, milk yield had a negative correlation with both involuntary and voluntary culling, while fat and protein percentages showed a positive correlation with involuntary culling and a negative correlation with voluntary culling, though none were significant (*p* > 0.05) (Table 8). In > 500‐head cow herds, milk yield and fat percentage had a negative correlation with both involuntary and voluntary culling, while protein percentage showed a positive correlation with involuntary culling and a negative correlation with voluntary culling, but none were significant (*p* > 0.05) (Table 8).

Table 8 aggregates involuntary and voluntary culling reasons. Comparing the two, we see that the trends observed in Table 8 for the largest herds (> 500 head) regarding voluntary culling (negative correlation with milk yield) are consistent with the strong negative correlation seen in Table 9 for low milk yield as a voluntary culling reason across all large herds. There is a document that provides insights into how farm characteristics, including herd size, influence management decisions related to culling. If it discusses the economic pressures or management strategies in different‐sized farms, those could indirectly explain some of the correlations observed in Table 8. For instance, larger farms might have stricter culling policies based on production efficiency [26]. Similarly, another paper provides context on culling practices, but without specific correlation data related to herd size and production, a direct comparison to the coefficients in Table 8 is not feasible [33].

**TABLE 9:** Correlation between production records and involuntary and voluntary culling reasons in herds with more than 500 cows between 2012 and 2014.

Involuntary culling reasons	Milk yield	Milk protein percentage	Milk fat percentage
Infectious disease	0.13	0.04	0.23
Noninfectious disease	0.05	−0.07	0.11
Mastitis	1.00^∗∗^	−1.00^∗∗^	−1.00^∗∗^
Udder disorders	−0.55	0.08	−0.96
Lameness	0.18	0.34	0.40
Dystocia (difficult calving)	—	—	—
Reproductive disorders	0.07	0.23	0.25
Mortality	—	—	—
Physical injury	−0.02	0.31	0.21

**Voluntary culling reasons**	**Milk yield**	**Milk protein percentage**	**Milk fat percentage**

Low milk yield	1.00^∗∗^	−1.00^∗∗^	−1.00^∗∗^
Aging	−0.55	0.08	−0.96
Financial constraints	—	—	—
Economic inefficiency	—	—	—
Surplus livestock	—	—	—
Unknown reasons	0.10	−0.02	0.18

**Involuntary culling reasons**	**Milk yield**	**Milk protein percentage**	**Milk fat percentage**

Infectious disease	−0.67	−0.34	−0.71
Non‐infectious disease	0.94	−0.75	0.92
Mastitis	−1.00^∗∗^	1.00^∗∗^	−1.00^∗∗^
Udder disorders	−0.55	0.01	−0.51
Lameness	0.78	−0.92	0.74
Dystocia (difficult calving)	—	—	—
Reproductive disorders	0.97	−0.67	0.96
Mortality	—	—	—
Physical injury	0.19	−0.96	0.14

**Voluntary culling reasons**	**Milk yield**	**Milk protein percentage**	**Milk fat percentage**

Low milk yield	−0.89	0.02	−0.91
Aging	−1.00^∗∗^	−1.00^∗∗^	−1.00^∗∗^
Financial constraints	—	—	—
Economic inefficiency	—	—	—
Surplus livestock	—	—	—
Unknown reasons	−1.00^∗∗^	−1.00^∗∗^	−1.00^∗∗^

*Note:* ^∗^ and ^∗∗^ show that correlation is significant at the 0.05 and 0.01 levels, respectively.

Langford and Stott [34] provide insights into whether production levels influence the decision to cull a cow early or late in her lactation or lifespan, potentially shedding light on the correlations seen in Table 8 across different herd sizes [34]. Yanga and Jaja [29] provide an overview of culling and mortality reasons. It might discuss how these factors vary with herd size, which could provide a broader context for interpreting the correlations in Table 8; for instance, larger herds might have different disease pressures or management strategies affecting both culling and mortality [29].

The data in Table 8 suggest that the relationship between production traits and culling decisions varies with herd size. In smaller herds (20–100), higher producing cows seem more likely to be involuntarily culled, a trend not observed in larger herds. Voluntary culling in midsized herds (101–200) appears to target cows with higher milk yield but lower fat and protein percentages. In larger herds (201–500 and > 500), voluntary culling shows a tendency to remove cows with lower milk yield. These differences could be attributed to various factors that often correlate with herd size, such as management intensity, disease prevalence, economic pressures, and culling strategies. Larger farms might have more stringent production targets and readily cull lower producing cows voluntarily, while smaller farms might be more tolerant or face different reasons for involuntary culling of higher producing animals.

Correlations between production parameters and specific culling reasons in 201–500 and > 500 head cow herds are shown in Table 9. In 201–500 head cow herds, infectious diseases had a positive but nonsignificant correlation with milk yield, fat percentage, and protein percentage (*p* > 0.05); besides, noninfectious diseases had a positive correlation with milk yield and fat and a negative correlation with protein (nonsignificant, *p* > 0.05). Mastitis had a negative correlation with fat and protein and a positive correlation with milk yield (significant at *p* < 0.01). Udder disorders showed a negative correlation with milk yield and fat percentage and a positive correlation with protein percentage (nonsignificant, *p* > 0.05). Lameness and reproductive disorders had a positive correlation with milk yield, fat percentage, and protein percentage (nonsignificant, *p* > 0.05). Physical injuries showed a positive correlation with milk fat and protein percentages and a negative correlation with milk yield (nonsignificant, *p* > 0.05).

The correlation coefficients of ±1.00 observed in Table 9 for mastitis and low milk yield likely reflect limited sample size in the > 500 head category (*n* = 6 farms) rather than perfect biological relationships. These values should be interpreted with caution and considered indicative of strong associations requiring validation in larger datasets.

The results of the correlation study between production records and voluntary culling reasons in dairy herds (201–500 cows) indicated that low milk yield had a negative correlation with fat and milk protein percentages but a positive correlation with milk production. This correlation was statistically significant at the 1% level (*p* < 0.01). Aging showed a negative correlation with milk yield and fat percentage but a positive correlation with protein percentage; however, these correlations were not significant (*p* > 0.05). Unknown culling reasons exhibited a positive correlation with milk yield and fat percentage but a negative correlation with protein percentage, though these values were not significant (*p* > 0.05) (Table 9).

In herds with over 500 cows, infectious diseases showed a negative correlation with milk yield, fat, and protein percentages, though these correlations were not significant (*p* > 0.05). Non‐infectious diseases had a positive correlation with milk yield and fat percentage but a negative correlation with protein percentage, which was also nonsignificant (*p* > 0.05). Mastitis exhibited a negative correlation with milk yield and fat percentage but a positive correlation with protein percentage, significant at the 1% level (*p* < 0.01). Udder disorders showed a negative correlation with milk yield and fat percentage but a positive correlation with protein percentage, though nonsignificant (*p* > 0.05). Lameness had a positive correlation with milk yield and fat percentage but a negative correlation with protein percentage, which was not significant (*p* > 0.05). Fertility issues showed a positive correlation with milk yield and fat percentage but a negative correlation with protein percentage, also nonsignificant (*p* > 0.05). Physical injuries had a positive correlation with milk yield and fat percentage but a negative correlation with protein percentage, though nonsignificant (*p* > 0.05) (Table 9). In herds exceeding 500 cows, low milk yield showed a negative correlation with milk production and fat percentage but a positive correlation with protein percentage, though nonsignificant (*p* > 0.05). Aging and unknown reasons exhibited a negative correlation with milk yield, fat, and protein percentages, with these correlations being significant at the 1% level (*p* < 0.01) (Table 9).

It was reported that the main reasons for culling were infertility and reproductive diseases (35.9%), diseases of the locomotor system (15.1%), udder diseases (13.1%), and metabolic and digestive diseases (12.9%) (Adam Oler et al., 2012). Table 9 shows a strong positive correlation between high milk yield and reproductive disorders and lameness (a locomotor system disease), suggesting that higher yielding cows in the larger herds studied might be more susceptible to these issues, leading to culling. The Oler et al. [27] study also noted a decrease in voluntary culling for low productivity, which contrasts with the strong correlation observed in Table 9 for low milk yield as a voluntary reason (Adam Oler et al., 2012). This discrepancy might be due to the different time periods (2006–2011 vs. 2012–2014) or the focus on high‐yielding cows in the Polish study versus a broader range in the data underlying (Table 9). Doncel‐Díaz et al. [4] focused on pasture‐based systems, which might have different dynamics compared to the larger herd sizes (> 500 cows) represented in Table 9. The reasons for culling and their association with production could vary due to different management practices and environmental factors. Without specific data from this study on correlations with production, a direct comparison is difficult [4].

### 3.7. Seasonal Effects on Production Records in Herds of 201–500 Cows and Over 500 Cows

Analysis of seasonal effects in herds of 201–500 cows revealed that winter had a higher mean of milk yield, fat, and protein percentages compared to summer, though the difference was not significant (*p* > 0.05) (Table 10).

**TABLE 10:** Effect of season on milk yield, fat percentage, and protein percentage in the studied dairy farms with different herd sizes (201–500 and > 500 head).

Season	Overall mean^1^		
	Milk yield (kg)	Milk protein percentage (%)	Milk fat percentage (%)
Summer	0.82 ± 30.46	0.03 ± 3.07	0.07 ± 3.08
Winter	0.66 ± 32.63	0.03 ± 3.18	0.07 ± 3.30

	**Milk yield (kg)**	**Milk protein percentage (%)**	**Milk fat percentage (%)**

Summer	0.82 ± 25.06	0.04 ± 3.03	0.05 ± 2.86^b^
Winter	1.92 ± 26.15	0.05 ± 3.14	0.09 ± 3.19^a^

*Note:* The means in the same column with different superscripts are significantly different at the 5% level (*p* < 0.05).

^1^The means are presented as mean ± standard deviation (SD).

In herds with over 500 cows, winter showed a higher mean of milk yield and protein percentage than summer, but the difference was not significant (*p* > 0.05). However, fat percentage was significantly higher in winter than in summer (*p* < 0.05) (Table 10). A study by Ozrenk and Inci [35] investigated the seasonal changes in cow milk composition. Their findings indicated that milk fat and nonfat dry matter contents were lowest during the summer months and highest during the winter months, while protein content also followed a similar trend, being lower in summer and higher in winter. They attributed these variations to factors such as environmental temperature and feeding regimes, noting a negative correlation between environmental temperature and milk fat and protein content [35].

A review of culling rates in multiple provinces identified reproductive disorders, nutritional and metabolic abnormalities, and infectious diseases as the three primary culling reasons [36]. In Khorasan Province, disease occurrence accounted for 47.14% of culling, followed by fertility issues (25.65%) and low production (7.01%). Additional reasons included cattle sales, mastitis, udder disorders, and limb defects [37].

In a study by Seddighi & Mohammadi [1] on 23 Holstein herds in Kalat‐e Nayshabur, low fertility (35%) was the leading culling cause, followed by digestive disorders (12.6%), nutritional issues (10.85%), mastitis (9.6%), and lameness (8.3%). Most culled cows were crossbred females, averaging 4 years old, with 5.5% dying, 7.22% slaughtered, and the rest sold [1].

Involuntary culling refers to the removal of cows from the herd due to reasons beyond the farmer’s direct control; instead, voluntary culling refers to the farmer’s decision to remove cows for reasons like low production, old age, or to improve herd genetics. In Iran (Fars Province), involuntary culling (74% of total culls) was primarily due to infertility (32.6%), mastitis (6.5%), postpartum complications (5.2%), left displaced abomasum (4.8%), physical injuries (4.2%), lameness (3.5%), and miscellaneous causes (8.7%) [38].

In Zanjan Province (Iran), fertility problems dominated culling (20.44% voluntary, 40.96% involuntary) (74Farsi). Similarly, Khorasan Razavi reported infertility, mastitis, low yield, and lameness as key culling factors [39]. In Tehran Province, recurrent abortion and infertility (29.4%) were the top culling reasons, followed by economic inefficiency (18%) and infectious diseases (15.2%). Other causes included metabolic disorders (9.8%), mastitis (7.3%), surplus stock (5.3%), injuries (5.1%), aging (3.6%), genetic defects (3.5%), financial constraints (2.1%), and dystocia (0.8%) (19). In traditional herds of Khorasan Razavi Province (in Iran), crossbred cows (88.99%) were culled more than native (6.76%) or purebred (4.25%) cattle. Lameness (70.93%) and milk fever (46.71%) were the leading culling reasons [36]. A 2009 study in Hashtgerd (Iran, Alborz Province) on 10 industrial dairy farms (3970 cows, 513 culled annually) classified culling into 12 categories: infectious diseases (21.64%), metabolic disorders (1.95%), reproductive issues (25.73%), unforeseen accidents (13.45%), postpartum disorders (8.38%), udder abnormalities (4.48%), lameness (3.31%), digestive disorders (7.41%), foreign bodies (3.12%), low production (3.7%), abortion (2.53%), and surplus stock (4.29%). Reproductive, infectious, and accidental causes were predominant [36].

In Tehran Province (Iran), common involuntary culling reasons included disease (10.33%), infertility (3.4%), mastitis (1.57%), dystocia (0.27%), lameness (1.88%), and genetic disorders (0.8%) [40]. Congleton and King [41] reported that extending lactation from 2.8 to 3.3 years increased profitability. Maximizing milk output requires cows to reach maturity, allowing selective culling of low‐yielders and reducing replacement costs [41].

Mastitis led to culling or early drying‐off in 7% of cases, with 36% experiencing prolonged milk loss (911 kg per lactation period). Persistent mastitis or severe production drops often resulted in culling [42]. Smith et al. [43] reported that the relative culling rate due to mastitis was higher in small herds compared to medium‐sized herds, and both left herd percentage (LHP) and relative left herd percentage (RLHP) increased with higher milk production. They also noted that the relative culling rate due to reproductive issues was higher in the southern United States compared to northern regions, attributing this primarily to the adverse effects of elevated heat and humidity. Additionally, their findings indicated that a greater proportion of cows were culled from small herds due to infertility [43].

Annually, approximately 5% of cows are replaced due to infertility and reproductive disorders, with low productivity and advanced age being the predominant reasons for culling [44]. Weigel et al. [45] observed that herds with larger labor forces, particularly family‐operated farms, exhibited lower involuntary culling rates for high‐producing cows due to enhanced care. Conversely, herds lacking facilities such as cooling systems, sprinklers, and dedicated calving areas experienced higher involuntary culling rates. Furthermore, they reported that high‐yielding cows bred exclusively via artificial insemination (AI) had a lower risk of involuntary culling compared to those bred through natural service, whereas the opposite trend was observed in low‐producing cows [45].

A 2006 report by the U.S. Department of Agriculture (USDA) indicated that among all culled dairy cows, 26.7% were removed due to reproductive disorders and infertility, 26.5% due to mastitis, and 22.4% due to suboptimal production [36]. Hadley et al. [46] conducted a study across multiple herds in 10 U.S. states from 1993 to 1999, analyzing culling rates and their causes. Their research classified culling reasons into nine primary categories, with results stratified by herd size and geographic location [46]. Ahlman et al. [47] concluded that udder disorders accounted for 24.7% of dairy cow culling, followed by low fertility (23.6%) and poor milk yield (8.8%) [47]. Approximately half of all culling events are disease‐related, with the impact varying by lactation stage. In the United States, conditions such as milk fever, retained placenta, displaced abomasum, ketosis, ovarian cysts, and mastitis significantly elevate culling risk at specific lactation phases [48].

Reproductive failure (8 cases), low production (6 cases), mastitis (5 cases), lameness (3 cases), downer cow syndrome (3 cases), peritonitis (2 cases), calving‐related disorders (2 cases), and unknown causes (2 cases) were the primary disease–related culling reasons [36]. Esslemont et al. [49] found that digestive and metabolic disorders accounted for only 1.2% of culling events. While this proportion is low, such disorders may indirectly contribute to metabolic diseases by disrupting mineral balance [49].

Multiple studies highlight that diseases can delay conception, prolong the days open period, and increase the likelihood. High‐production herds and thrice‐daily milking (vs. twice‐daily) exhibit higher involuntary culling rates for high‐yielding cows. Conversely, herds with dedicated calving facilities, cooling systems (e.g., fans, misters), and headlock stalls experience lower involuntary culling. Additionally, herds relying on purchased heifers face higher culling rates than those raising their own replacements. Full adoption of AI also reduces culling compared to natural mating or mixed breeding systems [36, 50, 51]. Ahlam reported that researchers analyzed culling causes in 13 Tehran City herds (average size: 792 head cows [dry and lactating]). They estimated culling probabilities for seven categories [36].

### 3.8. Estimated Culling Probability for Infectious Diseases

The culling probability for infectious diseases, based on estimated coefficients, peaks during the third lactation period and subsequently declines, reaching its lowest level by the sixth lactation or later. Spring exhibits the highest culling probability for both calving and culling seasons, followed by summer. The lowest culling probability was estimated for autumn calving and winter culling seasons. Regarding relative milk production, cows with lower production compared to their herdmates had a higher culling probability, while those with higher relative production also exhibited elevated culling risks. The culling probability was highest between 61 and 200 days postpartum.

### 3.9. Estimated Culling Probability for Locomotor Disorders

The culling probability for locomotor disorders was lowest in the third lactation period and highest from the sixth lactation onward. For calving and culling seasons, spring had the highest and lowest probabilities, respectively. The probability of calving leading to culling decreased toward autumn, while culling probability increased, reversing in winter. Cows with average relative milk production had the lowest culling risk, whereas higher producing cows faced greater culling probabilities. Cows between 61 and 200 days in milk had the highest culling risk, which decreased with shorter or longer production intervals.

### 3.10. Estimated Culling Probability for Respiratory and Cardiovascular Diseases

The culling probability for respiratory and cardiovascular diseases was highest in the first lactation and declined with subsequent lactations. A decreasing trend in culling probability was observed from the beginning to the end of the year for both calving and culling seasons. Higher relative milk production correlated with a linear increase in culling probability. The postpartum culling interval pattern mirrored that of locomotor disorders.

### 3.11. Estimated Culling Probability for Reproductive Disorders

Reproductive disorders had the highest relative culling probability among all categories, necessitating focused attention on high‐risk periods. Culling probability was low in the first lactation due to reduced susceptibility to reproductive culling factors, increased slightly until the fifth lactation, and rose sharply thereafter. Cows with intermediate milk production faced higher culling risks than herdmates. Culling probability declined until 200 days in milk but increased steeply thereafter, with nearly all culls beyond 400 days attributed to reproductive issues. Summer had the lowest culling probability, while autumn and winter had higher rates. Spring calving was associated with the highest probability of culling.

### 3.12. Estimated Culling Probability for Mastitis and Udder Disorders

Culling probability for udder disorders decreased until the third lactation, peaked in the fourth and fifth lactations, and then declined. Spring‐calving cows had the lowest culling probability, while autumn‐calving cows had the highest. For culling seasons, autumn and summer exhibited the lowest and highest probabilities, respectively. Cows with average relative production had lower culling risks, whereas higher producing cows faced greater risks. The highest culling probability occurred between 60 and 200 days postpartum.

### 3.13. Estimated Culling Probability for Digestive Disorders

For digestive disorders, culling probability was highest until the third lactation and subsequently declined. Autumn calving had the highest probability of leading to culling, while spring exhibited the opposite trend. Culling probability was associated with intermediate relative milk production. Cows between 8 and 200 days in milk had elevated culling risks.

### 3.14. Estimated Culling Probability for Metabolic and Nutritional Diseases

Culling probability for metabolic and nutritional diseases increased with lactation number, except for a slight dip in the third lactation. Seasonal variations in calving‐related culling were minimal, with winter showing the lowest probability of calving leading to culling and autumn having the lowest overall culling probability. Spring had the highest culling probability. Contrary to expectations, higher producing cows had lower culling risks, while average production levels were associated with the highest culling probability. Culling probability decreased with longer production intervals [36].

### 3.15. Herd Size and Culling Patterns

This study demonstrates a direct relationship between herd size (a proxy for management intensity) and involuntary culling rates. Larger herds had reduced individual oversight, leading to higher culling rates. In small herds (20–100 cows), owner‐managers provided closer supervision, resulting in stable milk production and fewer involuntary culls. The primary culling reason in these herds was infertility (8.33 ± 6.89%), with the lowest overall culling rates. In contrast, herds exceeding 500 cows had infectious and metabolic diseases as the leading culling causes, followed by reproductive issues. Economic analyses suggest an optimal herd size of 201–500 cows for this region (and potentially nationally), as these herds achieved superior milk yield (averaging 32 kg/day with 3.1% fat) and lower culling rates for mastitis, udder disorders, and lameness compared to larger herds. In herds > 500 cows, diseases (infectious and noninfectious) were the primary culling reasons, aligning with prior studies [36, 38, 47, 52]. Despite infertility being the dominant culling factor in smaller herds, the elevated culling rates in large herds necessitate tailored strategies. Herds should adopt breeding plans aligned with their specific culling patterns and avoid ad hoc semen purchases, which may exacerbate economic pressures and involuntary culling. Economic profitability analyses should guide strategic decisions for herd managers.

The relationship between herd size and culling patterns observed in this study is consistent with findings from other dairy systems worldwide. Recent research has demonstrated that herd size affects both the prevalence of health disorders and the economic viability of dairy operations [19, 20]. In larger herds, the reduced ability to provide individual animal care may contribute to higher rates of health‐related culling, while smaller herds may face economic pressures that lead to increased voluntary culling for low productivity [21].

Several methodological limitations should be considered when interpreting these findings. First, the convenience sampling approach may limit generalizability to farms with less comprehensive record‐keeping. The 27 farms included represent only 71% of eligible farms, and farms with better records may systematically differ in management quality and culling patterns [15]. Second, the “unknown” category for voluntary culling (57.27% in 2010, decreasing to 6.14% in 2014) indicates variable data quality over the study period, potentially underrepresenting certain culling reasons in earlier years. Third, the correlational design prevents causal inference; associations between production traits and culling should not be interpreted as causative [10]. Fourth, the small sample size in the > 500 head category (*n* = 6 farms) limited statistical power for subgroup analyses, as reflected in the extreme correlation coefficients observed. Despite these limitations, the comprehensive dataset across 5 years and multiple herd sizes provides valuable insights into Tehran’s dairy culling patterns.

Regarding our study hypothesis, the findings partially support the proposed relationships. The hypothesis that culling reasons would differ significantly across herd sizes was confirmed, with larger herds exhibiting higher involuntary culling rates for health‐related issues. The second hypothesis—that milk production traits would show strong negative correlations with fat and protein percentages—was supported by the observed correlation coefficients (−0.79 to −0.89 for fat; −0.77 to −0.88 for protein). However, the third hypothesis regarding the optimal balance in 201–500 head herds was only partially supported, as these herds showed favorable productivity but also faced significant reproductive disorders requiring targeted management interventions [18, 19].

The findings of this study have several practical implications for dairy herd management in Tehran Province. First, the identification of 201–500 head as an optimal herd size suggests that farm expansion beyond this threshold may lead to diminishing returns due to increased health‐related culling. Second, the strong association between reproductive disorders and involuntary culling underscores the need for improved fertility management, including systematic reproductive monitoring and targeted breeding programs [20]. Third, the seasonal effects observed (higher milk yield and fat percentage in winter) indicate that heat stress mitigation strategies are essential during summer months to maintain productivity and reduce culling risk [7]. Finally, the decreasing trend in “unknown” culling reasons over the study period highlights the value of improved record‐keeping for data‐driven decision‐making [19].

## 4. Summary of Results, Practical Conclusions, and Strategic Suggestions

This study demonstrates that involuntary culling (70%–76%) predominates in Tehran’s dairy farms, with reproductive disorders (15%–24%), mastitis (13%–18%), and lameness (11%–15%) as the primary causes, consistent with global dairy culling patterns [9, 30]. Herd size significantly influences culling dynamics: Larger herds (> 500 head) achieved higher milk yield (8750 kg) but experienced elevated involuntary culling for health issues, while smaller herds (20–100 head) showed stable production but higher relative voluntary culling due to low yield. Herds of 201–500 head exhibited an optimal balance between productivity (mean 32 kg/day) and lower culling rates for major health disorders. The negative correlations between milk yield and solids concentration (−0.79 to −0.89 for fat; −0.77 to −0.88 for protein) confirm the physiological trade‐off between volume and components, which intensifies in larger herds [22].

Practical recommendations include (1) implementing fertility‐focused breeding programs to reduce reproductive culling, potentially decreasing involuntary culls by 10%–15% [9]; (2) adopting herd‐size‐appropriate management strategies, with 201–500 head as an optimal target for Tehran’s industrial context; (3) enhancing record‐keeping to reduce “unknown” culling reasons and enable data‐driven decision‐making [15]; and (4) developing seasonal management protocols, as winter months showed significantly higher fat percentage and milk yield in large herds (*p* < 0.05), indicating heat stress mitigation is critical in summer. These strategies could extend productive herd life from 2.8 to 3.3 years, reducing replacement costs and improving sustainability.

### 4.1. Innovative Aspects

This investigation stands out for its detailed stratification by herd size (20–100, 101–200, 201–500, > 500 head), revealing nuanced relationships between production traits and culling reasons that are often overlooked in broader studies. For instance, while milk yield increased with herd size (from 6955 kg in small herds to 8750 kg in large herds), fat and protein percentages declined (4.06% to 3.86% for fat; 3.28% to 3.20% for protein), with strong negative correlations between yield and components (−0.79 to −0.89 for fat; −0.77 to −0.88 for protein) and positive correlations between fat and protein (0.91–0.94). These patterns underscore physiological trade‐offs in high‐production systems, where intensified management may dilute milk solids, impacting animal welfare and economic outcomes.

A key innovation is the emphasis on involuntary culling’s dominance (70%–76% of total culls), driven primarily by reproductive disorders (15%–20%), mastitis (13%–15%), and lameness (15%), with low milk yield varying by herd size. This builds on existing literature, such as Mohammadi and Sedighi [1], who reported similar health‐driven culling in Tehran farms, but diverges by linking these to herd‐scale dynamics—for example, larger herds exhibited stronger negative correlations, suggesting heightened stress from intensive operations [1]. Unlike global studies emphasizing low fertility as a universal issue [2], our data highlight region‐specific economic factors in voluntary culling (24%–30%), such as financial constraints in larger herds, contributing novel understanding of how local management challenges exacerbate welfare issues. This aligns with recent Iranian research, where poor fertility accounted for 34.9% of culls in Neyshabur herds, but extends it by proposing integrated breeding solutions tailored to Tehran’s industrial context.

Furthermore, the study’s integration of seasonal effects—higher winter yields and fat percentages in mid‐to‐large herds—innovates by connecting environmental stressors to production and culling, diverging from theories focused solely on genetic selection [5]. These insights enhance animal welfare by advocating proactive health monitoring, potentially reducing economic losses from premature culling, estimated at substantial replacement costs in high‐turnover systems [4].

### 4.2. Optimal Herd Size

Based on the data, an optimal herd size for balancing effective management, low culling rates, and profitability appears to be 201–500 head. These herds achieved the highest average milk yield (approximately 32 kg/day with 3.1% fat) while maintaining lower involuntary culling rates for key issues like mastitis, udder disorders, and lameness compared to > 500‐head herds. In contrast, smaller herds (20–100 head) had stable production but higher relative voluntary culling due to low yield, while larger herds (> 500 head) faced elevated health‐related disposals, aligning with increased culling as herd size grows [46].

This recommendation is supported by literature indicating that midsized herds optimize operational efficiency, spreading fixed costs without the disease pressures of overcrowding [43]. For farmers, aiming for 201–500 head could minimize culling (total removal rates averaged 37.6% in similar regions) while maximizing returns, as larger operations often incur higher involuntary losses due to diluted individual care. Economic models suggest this size reduces replacement needs by 4%–5% per 2% culling increase, enhancing sustainability (Managing Heifer Inventories, 2025).

Additionally, here is a text‐based infographic summarizing key correlations across herd sizes (negative between yield and components, positive between fat and protein), derived from the study’s Table 2 and summary ranges:

• Correlations overview (ranges across herd sizes)
• Yield vs. fat: strongly negative (−0.79 to −0.89)
• Yield vs. protein: strongly negative (−0.77 to −0.88)
• Fat vs. protein: strongly positive (0.91 to 0.94)

### 4.3. Main Findings and Practical Applications

Key outcomes reveal involuntary culling’s predominance, with reproductive issues as the top factor across sizes, exacerbated in larger herds where noninfectious diseases also rise. Production correlations highlight dilution effects, and seasonal variations show winter advantages in yield and fat.

Practically, farmers should adopt tailored breeding programs selecting for fertility and disease resistance (e.g., AI over natural service to reduce culling by 20%–30%), improved nutrition plans balancing high yield with solids (e.g., via heat‐stress mitigation in summer), and health monitoring systems like electronic tracking to detect issues early, potentially cutting involuntary culls by 10%–15% [53, 54]. These innovations aim to extend cow longevity from 2.8 to 3.3 years, boosting productivity and reducing costs.

### 4.4. Broader Implications

Beyond Tehran, these findings imply benefits for global dairy cattle farmers, wherever high culling erodes dairy industry sustainability. Importantly, the results of this article are not limited to Iran and hold significant global relevance; they can be applied universally in the dairy cattle industry worldwide to optimize herd size, minimize culling rates, and ultimately increase profitability by addressing common physiological and management challenges shared across intensive farming systems in diverse regions. Reducing involuntary culling rates through better welfare could lower environmental impacts (e.g., fewer replacements mean reduced methane emissions) and enhance economic resilience amid market volatility. In similar arid regions, policies promoting midsized herds and fertility‐focused breeding could foster regulations for health standards, ensuring long‐term viability [3].

### 4.5. Future Research Directions

Future studies should incorporate technological advancements, such as precision farming tools for real‐time health monitoring, to validate interventions longitudinally. Exploring genomic selection for resilient traits or climate‐adapted breeds could address seasonal effects, while comparative analyses with modern data (post‐2014) would assess evolving trends in Iran’s dairy sector.

## Funding

The preparation of this manuscript did not receive financial assistance.

## Ethics Statement

This study was conducted using retrospective data from farm records and did not involve any direct animal experimentation, invasive procedures, or interventions that could affect animal welfare. All data were obtained from existing farm records with the informed consent of participating farm owners. According to the institutional guidelines and national regulations governing research on farm animals, formal ethical committee approval was not required for this type of retrospective observational study using existing farm records. However, the study was conducted in accordance with the ethical principles for animal research and followed the guidelines for responsible conduct of research. The authors confirm that all participating farms were informed about the study objectives and provided voluntary consent for data access and use.

## Conflicts of Interest

The authors declare no conflicts of interest.

## Data Availability

The data that support the findings of this study are available from the corresponding author upon reasonable request.
